# Smart Helmet-Based Proximity Warning System to Improve Occupational Safety on the Road Using Image Sensor and Artificial Intelligence

**DOI:** 10.3390/ijerph192316312

**Published:** 2022-12-06

**Authors:** Yeanjae Kim, Yosoon Choi

**Affiliations:** Department of Energy Resources Engineering, Pukyong National University, Busan 48513, Republic of Korea

**Keywords:** workforce safety, smart helmet, personal proximity warning system, image sensor, artificial intelligence

## Abstract

Recently, collisions between equipment and workers occur frequently on the road in construction and surface mining sites. To prevent such accidents, we developed a smart helmet-based proximity warning system (PWS) that facilitates visual and tactile proximity warnings. In this system, a smart helmet comprising an Arduino Uno board and a camera module with built-in Wi-Fi was used to transmit images captured by the camera to a smartphone via Wi-Fi. When the image was analyzed through object detection and a heavy-duty truck or other vehicle was detected in an image, the smartphone transmitted a signal to the Arduino via Bluetooth and, when a signal was received, an LED strip with a three-color LED visually alerted the worker and the equipment operator. The performance of the system tested the recognition distance of the helmet according to the pixel size of the detected image in an outdoor environment. The proposed personal PWS can directly produce visual proximity warnings to both workers and operators enabling them to quickly identify and evacuate from dangerous situations.

## 1. Introduction

According to the US Bureau of Labor Statistics, 27% of the 1083 construction and mining casualties in the US in 2017 was related to collisions with equipment [1]. A report stated that 138 accidents due to collisions have occurred at surface mines in Western Australia since 2015 [2]. Proximity warning systems (PWSs) have been developed to prevent such equipment collisions in construction and mining sites [3]. PWSs provide visual and/or audible proximity warnings to equipment operators, approaching pedestrians, or to other equipment within a specified distance [4].

Many researchers have developed PWSs to prevent such collisions in construction and surface mining sites. Ruff [5] investigated the detection of obstacles in a driver’s blind spot using a variety of sensors attached to a 50-ton dump truck. Also, based on the experimental results, the reliability and false-alarm rate of proximity detection technology using sensors were analyzed [6]. Ruff and Hession-Kunz [7] developed a PWS using a radio frequency identification system (RFID) and analyzed the problems through performance experiments. Schiffbauer [8] proposed a PWS for an equipment operator approaching a continuous miner. A wire loop antenna was installed on the continuous miner to generate a magnetic field, and a receiver measured the magnetic field strength. Visual, audible, and vibration alerts were generated to the equipment operator when a specific threshold was exceeded. Ruff [9] performed a performance test of a PWS using radar-based proximity detection technology. Ruff and Holden [10] developed a PWS using a global positioning system (GPS) and peer-to-peer communication. In addition, comparative experiments were performed on various proximity detection sensors that can be used for the development of PWSs [11,12]. Baek and Choi [13] proposed a Bluetooth low energy (BLE) beacon-based PWS that provides proximity alerts to equipment operators using a smartphone. BLE beacons were attached to the mining equipment and dangerous areas, and a smartphone was mounted on the equipment. Table 1 summarizes the characteristics of sensor technology that have been traditionally used for PWS development.

Recently, researchers have proposed a variety of wearable PWSs to provide proximity warnings to pedestrians. Unlike conventional PWSs, a signal transmitter is installed on the device, and the receiver is attached to the pedestrian. Wearable PWSs can be categorized into worn-type personal alarm devices (PADs), smart vests, and smart glasses. PADs can be worn on the worker’s belt, pocket, and cap and mainly provide audible or vibration alerts [15]. Examples of commercialized PAD products include ELOshield by ELOKON [16], the SmartZone proximity system by Komatsu and JoyGlobal [17,18], and HazardAvert^®^ by SRATA [19,20,21,22,23]. Smart vests provide vibration alerts using haptic or tactile sensors [24,25]. Smart glasses measure the received signal strength indicator (RSSI) of a BLE signal transmitted from a Bluetooth beacon attached to the device and provide a proximity warning when the RSSI exceeds a specified threshold [26].

A smart helmet is another type of device that could be used as a wearable PWS. Recently, a smart helmet-based PWS was developed using BLE technology [15]. Mining equipment, such as dump trucks, excavators, and loaders, had Bluetooth beacons, and pedestrian workers recognized the BLE signal from the beacons using smart helmet-based PWSs. Therefore, the smart helmet generated a visual proximity warning using light-emitting diode (LED) lights when mining equipment approached a pedestrian worker. Using the LED lights, both the machine operators and pedestrians on the road could recognize proximity warnings. However, a limitation of this system is that it provides proximity warnings to only those devices with a Bluetooth beacon installed. If equipment without a Bluetooth beacon is put into the mine site, the smart helmet-based PWSs could not provide a proximity warning to the workforce. So far, a smart helmet-based PWS has not yet been developed that generates proximity warnings to a workforce by recognizing unspecified equipment to which a sensor such as a Bluetooth beacon is not attached. To overcome this limitation, we developed a smart helmet-based PWS using an image sensor rather than a Bluetooth beacon.

The objective of this study is to develop a smart helmet-based PWS that facilitates visual and tactile proximity warnings to workers and operators at surface mining sites. The smart helmet PWS collects image data from a camera module that is analyzed in a cloud server using an artificial intelligence (AI) technology to determine the proximity of a hazardous object. When this object is approaching, the LED light turns on and a vibration signal is sent to the smartphone to provide a hazard warning. The smart helmet PWS’s recognition distance was analyzed in this study according to the set minimum diagonal length, and its hazard warning accuracy was evaluated according to the face angle between the camera module and the mining equipment. 

## 2. Methodology

### 2.1. Materials and Methods

**Figure 1:**
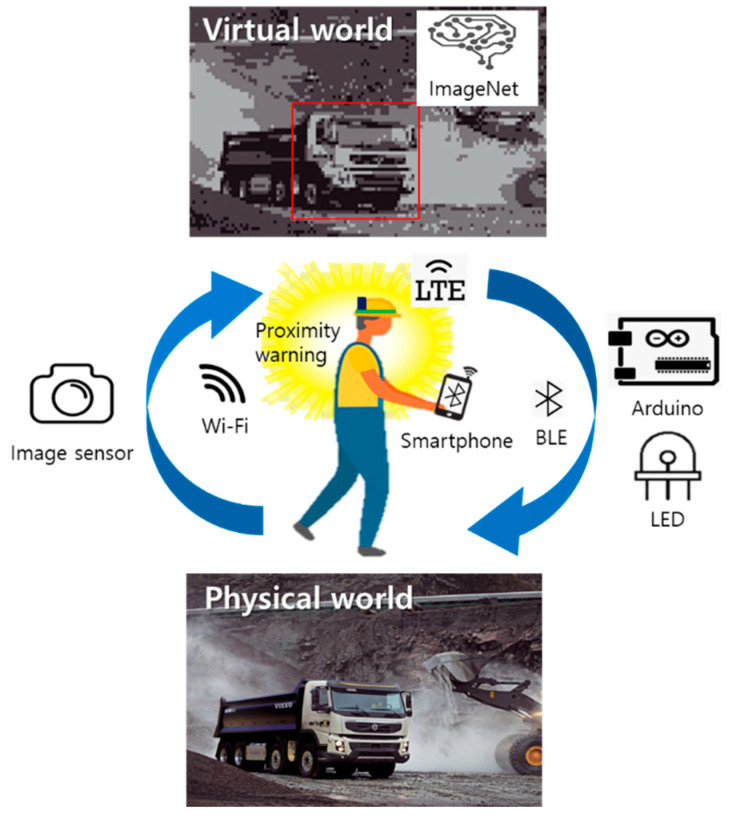
Overview of the AI-based smart helmet proximity warning system.

Figure 1 illustrates the concept of the smart helmet-based PWS using an image sensor and an AI approach. The smart helmet worn by a worker acquires images from the ESP32-CAM and transmits them to a smartphone via Wi-Fi. This image is then sent to a cloud server using an open application program interface (API), and the result based on the object recognizer is received from the server. When a hazardous object, such as a truck or a similar vehicle, is detected in the object recognition data, the smartphone sends a signal to the Arduino via Bluetooth. When the Arduino receives the Bluetooth signal, it sends a vibration signal to the smartphone and turns on the LED strip with the three-color LED to visually alert the operator. To detect hazards that approach from the rear outside of the worker’s field of vision, the ESP32-CAM module is attached to the back of the smart helmet. The smart helmet wearer receives a tactile proximity warning via the smartphone’s vibration actuator, which facilitates a swift detection and response to a dangerous situation. The visual proximity warning is relayed via the LED lights enabling both the helmet wearer and operator to quickly detect and respond to the dangerous situation. By setting a variety of hazardous objects, the system can be applied to different sites, such as general urban centers, mining sites, and construction sites. 

#### 2.1.1. Development of BLE Transmitter Using ESP32-CAM

**Figure 2:**
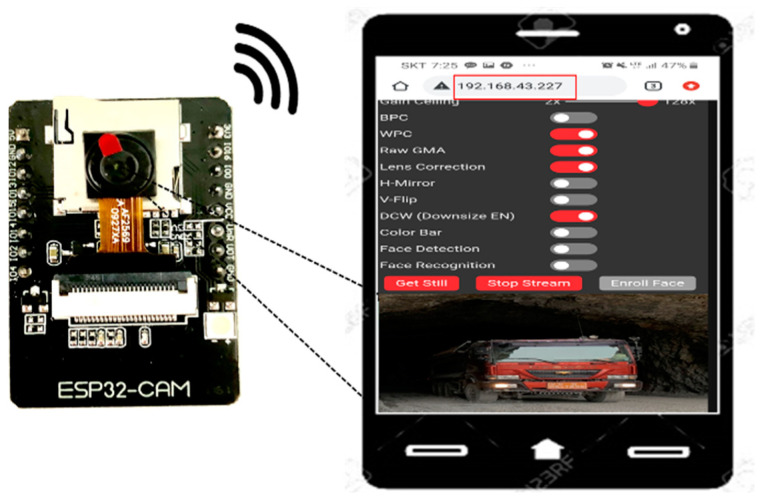
Operation process of the ESP32-CAM.

In the ESP32-CAM module [27], the Wi-Fi function and camera are integrated into one board, and an SD card slot is attached, which can be used to cheaply and easily develop CCTV recorders, video streaming devices, video transmission devices, and similar devices. To use the ESP32-CAM module, after connecting to a desktop using the future technology devices international (FTDI) programmer, the development environment was constructed using the ESP32 board manager in the Arduino’s integrated development environment (IDE), and the operation code was uploaded. After the code was uploaded, power was supplied to the ESP32-CAM, and it was used as a video streaming server. As shown in Figure 2, the images of the ESP32-CAM are received in real-time on the smartphone webpage via Wi-Fi.

**Figure 3:**
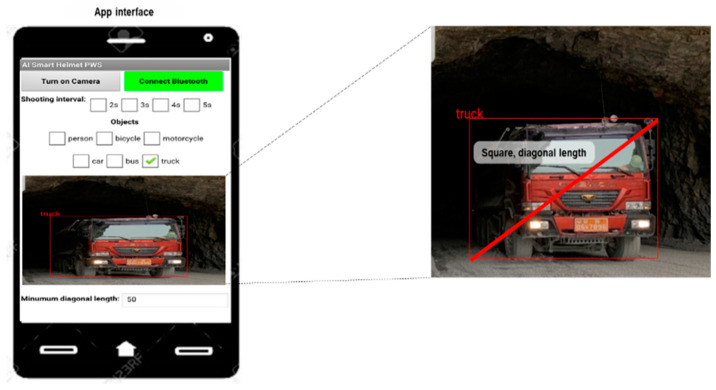
Interface and operating principles of the AI smart helmet PWS.

To analyze the images from the ESP32-CAM, we developed a smartphone application called AI Smart Helmet PWS. Figure 3 shows the interface and operating principles of the AI Smart Helmet PWS. When the “Connect Bluetooth” button is pressed, it connects with the Bluetooth module of the Arduino and, when the “Turn on Camera” icon is pressed, the video transmitted from the ESP32-CAM is saved as JPG images at 2 s intervals, converted to Base64 code, and displayed on the canvas. The converted Base64 code is transmitted to the public AI open API platform provided by the Electronics and Telecommunications Research Institute (ETRI) [28].

The public AI open API platform provides the object detection service based on a class-wise ensemble machine learning model named Rank of Experts [29]. The Rank of Experts model decomposes an intractable problem of finding the best detections for all object classes into small subproblems of finding the best ones for each object class. Then, the detection problem is solved by ranking detectors in order of the average precision rate for each class. The Rank of Experts model won 2nd place in the ILSVRC 2017 object detection competition [29].

In the smartphone application, the user can set hazardous objects using the checkboxes below “Objects”. If the image contains a set hazardous object, then a red box and the object’s name are displayed on the image. If the diagonal length of the red box is greater than the length entered in the text box next to “Minimum diagonal length”, then a Bluetooth signal is transmitted from the smartphone to the Arduino.

#### 2.1.2. Development of Receiver

**Figure 4:**
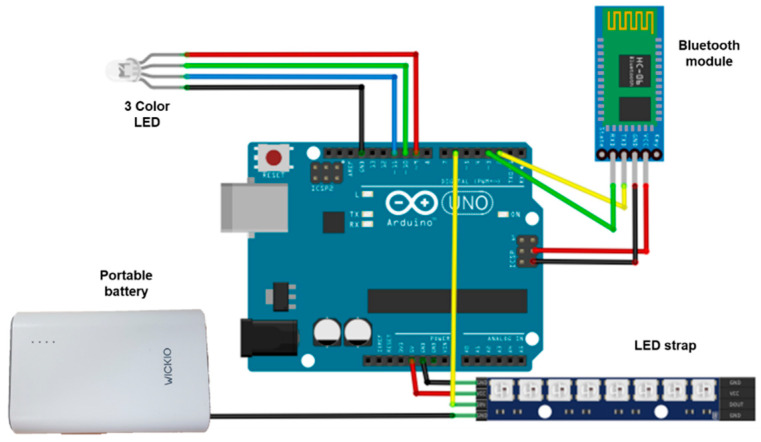
Configuration of circuits connected to the Arduino Uno board.

The Arduino UNO board reads the inputs such as light from sensors and button presses and converts them to outputs that operate motors, turn on LEDs, etc. The board can be instructed to perform different tasks by sending an instruction set to its microcontroller [30]. The HC-06 Bluetooth module is inexpensive, consumes very little power, and can interface with almost any controller or processor because it uses the UART interface [31]. To receive the Bluetooth signal transmitted from the smartphone, the smart helmet receiver used in this study was designed by combining the HC-06 Bluetooth module, Arduino Uno board, LED strip, and three-color LED. As part of the approach, a structural diagram was visualized based on the circuit diagram in Figure 4.

**Figure 5:**
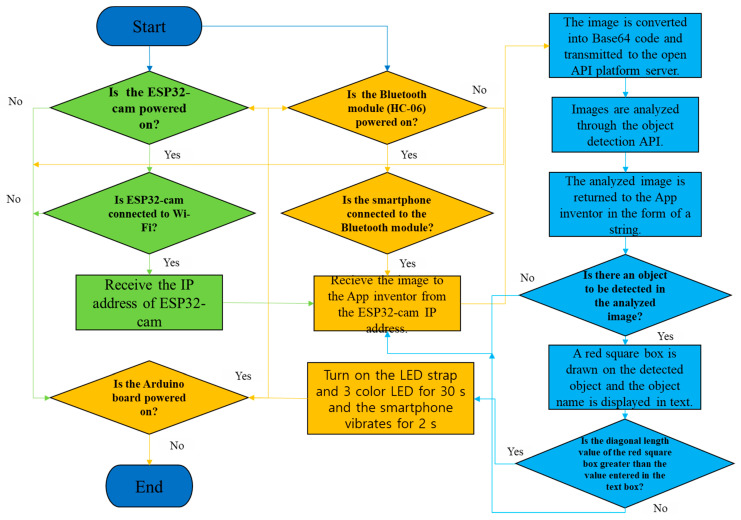
Operation algorithm of the AI smart helmet-based collision prevention system.

Figure 5 shows the algorithm for the overall process in which the receiver of the AI smart helmet receives the Bluetooth signal and generates a hazard warning. The principle of the algorithm is as follows:

(a) The video captured by the ESP32-CAM is loaded onto the smartphone via Wi-Fi. The acquired video is saved as JPG images in real time, and the saved images are converted into Base64 code using the smartphone app.
(b) The converted code is sent to the public AI open API server via HTTP and analyzed using the object detection learning model.
(c) When the processing for object detection is completed on the server, the analyzed information is returned as java script object notation (JSON) text data via an HTTP response message to the AI Smart Helmet PWS.
(d) An analysis is performed on the smartphone to determine whether the object information sent from the server contains any user-set hazardous objects. If such an object is detected, it is enclosed by a red box, and its name is displayed on the smartphone.
(e) When a hazardous object enters the hazard area, the smartphone sends a Bluetooth signal to the smart helmet, which turns on the LED strip and three-color LED and vibrates the smartphone to provide a hazard warning.

Pedestrian workers or operators who wear the helmet receive tactile and visual warnings, and nearby operators or workers are visually alerted by the LED strip, thereby facilitating quick detection and response to the dangerous situation.

#### 2.1.3. Recognition Distance Experiment According to Input Minimum Diagonal Length

**Figure 6:**
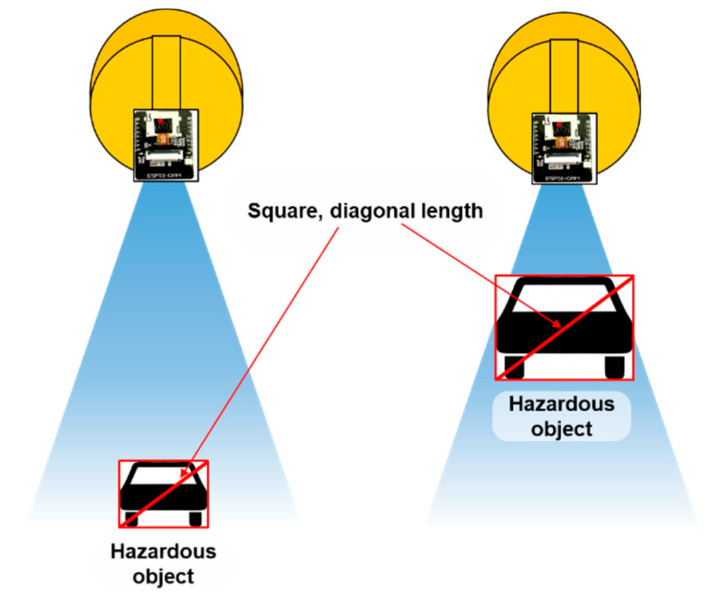
Overview of the experiment to measure the recognition distance according to input minimum diagonal length.

Assuming that the helmets will be used by riders of two-wheeler vehicles and personal mobility devices, the experiment was performed on a straight road within a university campus with the smart helmet worn by the rider of an electric scooter. As the electric scooter traveled at a speed of 10 km/h, a test vehicle moved toward the helmet wearer at a speed of 30 km/h from a distance of 100 m. We then measured the recognition distance at which the test vehicle was detected by the ESP32-CAM attached to the rear of the smart helmet. During the experiment, the minimum diagonal lengths for the AI Smart Helmet PWS app were set at 25-pixel intervals from 25 to 125 pixels (Figure 6), and 10 measurements were acquired for each input value. It should be noted that the minimum diagonal length was determined by counting the number of diagonal pixels in the lower left and upper right corners of the red rectangle drawn on the smartphone screen when the vehicle was recognized.

### 2.2. Experimental Measurement of Hazard Warning Accuracy 

**Figure 7:**
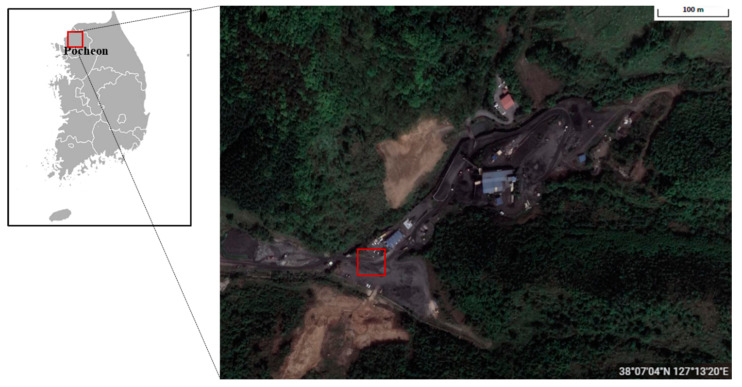
View of the experimental site.

We conducted an experiment based on the assumption of use by a mining site worker to evaluate whether the developed AI smart helmet could provide additional safety when used by field workers at construction or mining sites. The field experiment was performed at a Samyang Resources iron mine in Pocheon-si, Gyeonggi-do, South Korea (38°07′04″ N, 127°13′20″ E) (Figure 7).

**Figure 8:**
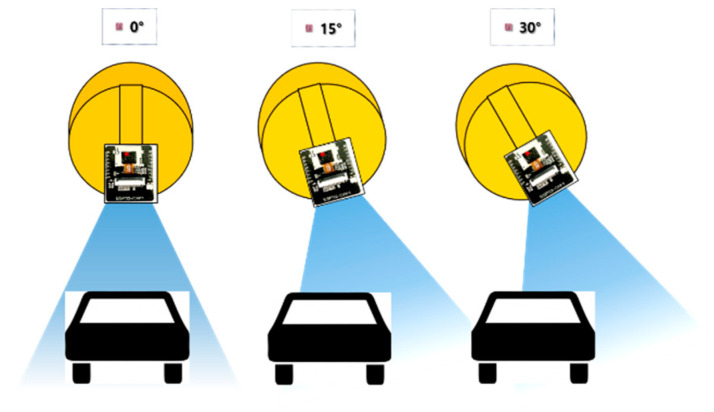
Overview of the experiment to measure the hazard warning accuracy according to the face angle between the camera module and hazardous object.

Assuming that the mining equipment in question approaches the field worker from the rear, the hazard area between the worker and mining equipment was set to a distance of 20 m. We investigated whether the hazard warning was activated before the mining equipment reached the hazard area when approaching the helmet wearer (experimental subject) from the rear at a speed of 20 m/s. The minimum diagonal length was set to 50 pixels. Using three face angles of 0°, 15°, and 30°, we conducted the experiment 10 times for each face angle between the subject’s smart helmet and the approaching mining equipment (Figure 8). Angles over 30° could not be tested since the mining equipment did not enter the camera module’s field of view when approaching the set hazard area. The parameters used to evaluate the accuracy were set as “True-positive”, “False-negative”, and “Recall”. 

## 3. Results

**Figure 9:**
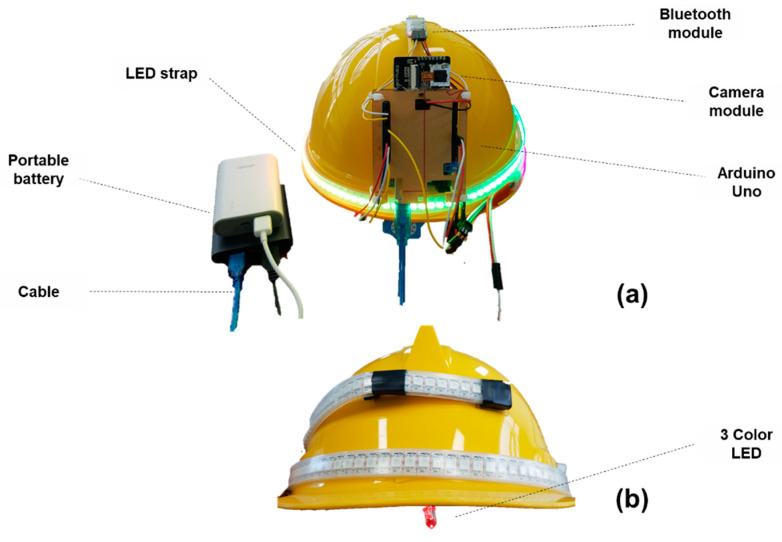
Overview of the AI smart helmet unit. (**a**) Oblique view; (**b**) Side view.

An AI smart helmet was developed using an Arduino Uno board, a camera module (ESP 32-CAM), a Bluetooth module (HC-06), an LED strip, and a three-color LED with a hard hat worn by field workers. Power was supplied via a portable battery. Figure 9a,b shows the front and rear of the device’s exterior, respectively.

**Figure 10:**
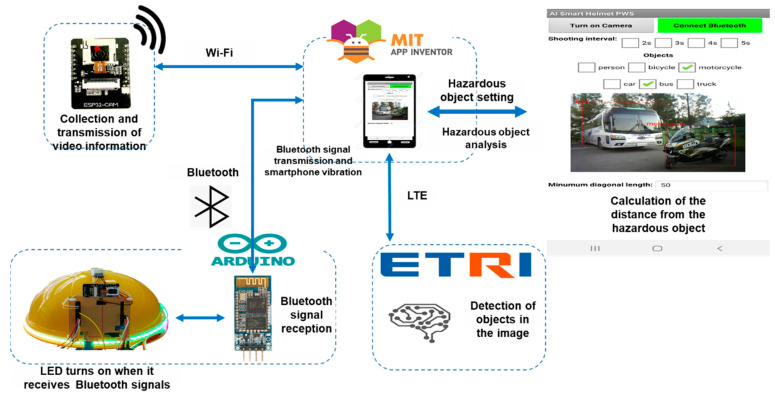
Conceptual diagram of the AI smart helmet-based PWS.

Figure 10 presents an overview of the developed AI smart helmet-based PWS system. Image information was acquired from the ESP32-CAM module and transmitted to the smartphone via Wi-Fi, and the acquired images were analyzed using the developed app on the smartphone. An object detection learning model was used to analyze the image data. Using this information, when a hazardous object approached the helmet wearer and reached the hazard area, a hazard warning was generated. This warning was in the form of tactile feedback that was produced in response to a vibration signal from the smartphone, and a Bluetooth signal was transmitted to the AI smart helmet. When the Bluetooth module (HC-06) attached to the AI smart helmet received the signal, it produced a visual warning via the LED strip and the three-color LED. Thus, using the LED visual warning and smartphone vibration tactile warning, not only the helmet wearer but also nearby operators and workers can recognize the hazard beforehand and quickly prevent an accident from occurring.

**Figure 11:**
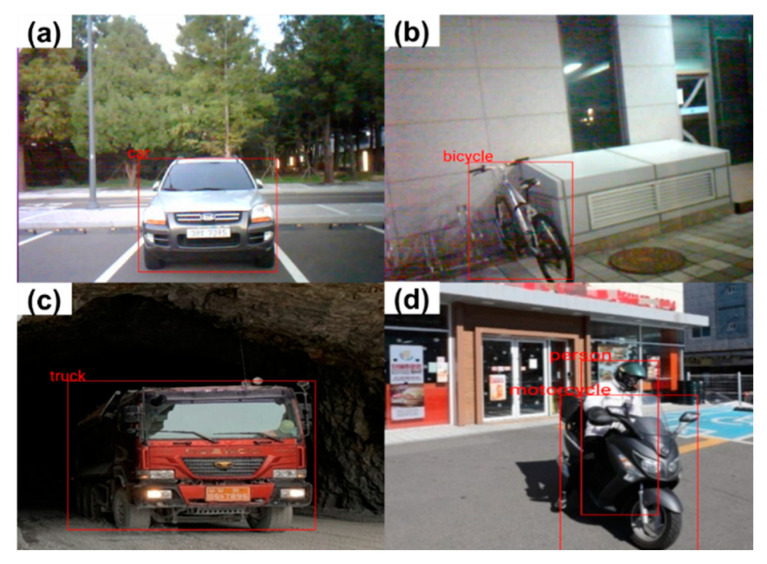
Hazardous object recognition screen in the AI smart helmet PWS app. (**a**) Car; (**b**) Bicycle; (**c**) Truck; (**d**) Motorcycle with person.

To provide an appropriate warning when a hazardous object approaches the helmet wearer by analyzing the image data, we developed an AI Smart Helmet PWS for Android OS. Six types of hazardous objects can be set in the app: person, bicycle, motorcycle, car, bus, and truck. Figure 11 shows that the app can recognize different hazardous objects. Given that the user can set various hazardous objects, the helmet can be used in a variety of environments, such as urban centers, mining sites, and construction sites.

**Figure 12:**
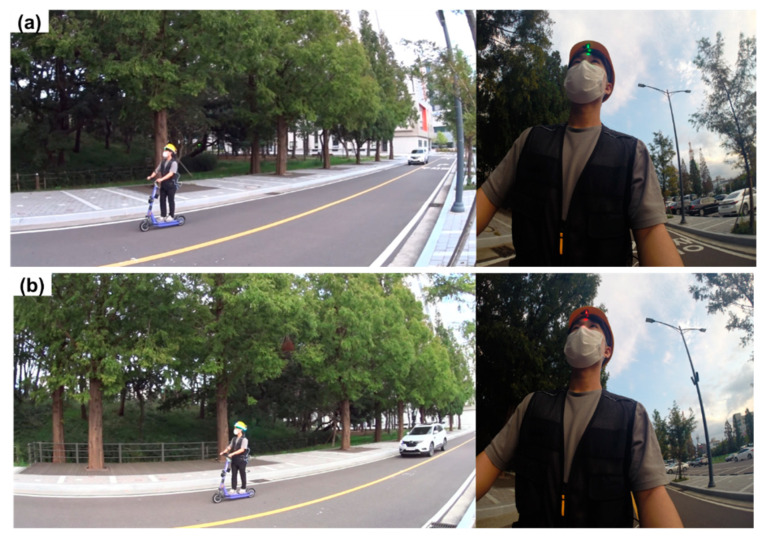
Visual warning via the LED strip and the three-color LED from the AI smart helmet. (**a**) Helmet wearer before the vehicle reaches hazardous area; (**b**) Helmet wearer when the vehicle reaches the hazardous area.

Figure 12 shows the results from the recognition distance experiment according to the input minimum diagonal length. A hazard warning is not produced until a vehicle that approaches the helmet wearer from the rear enters the recognition distance range (Figure 12a). A warning is generated when the vehicle enters the recognition distance range of the AI smart helmet (Figure 12b). Table 2 shows the main statistics for the recognition distance of the AI smart helmet according to the input minimum diagonal length. The average recognition distance was 63.25 m at 25 pixels, 24 m at 50 pixels, 14.25 m at 75 pixels, 11 m at 100 pixels, and 6.25 m at 125 pixels. Hence, as the minimum diagonal length increased, the recognition distance of the AI smart helmet decreased. In all experiments, when a vehicle came within detection range, the AI smart helmet detected the vehicle and provided a proximity alert in real time.

**Figure 13:**
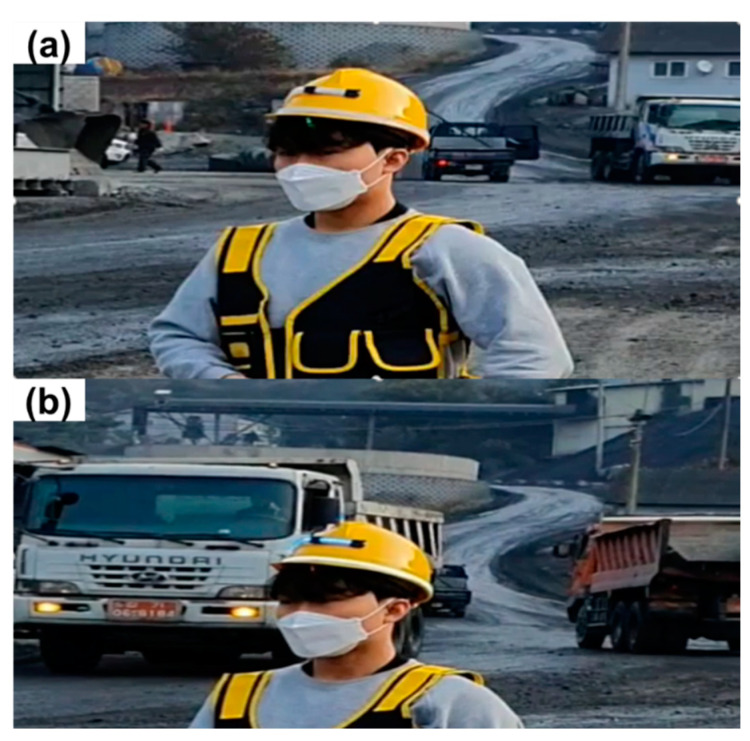
Visual warning provided by the AI smart helmet. (**a**) Before the mining equipment reaches the hazardous area; (**b**) When the mining equipment reaches the hazardous area.

Figure 13 shows the accuracy of the evaluation results of the hazard warning according to the face angle between the camera module and the mining equipment. The AI smart helmet does not produce a hazard warning before the mining equipment reaches the hazardous area (Figure 13a). A warning is generated when the mining equipment reaches the hazardous area (Figure 13b).

Table 3 shows the statistics for the accuracy of the evaluation of the AI smart helmet hazard warning according to the face angle between the camera module and the mining equipment. When the face angle between the camera module and mining equipment was 0° and 15°, the helmet produced a positive hazard warning in all 10 rounds of the experiment, and no false hazard warning was observed. At a face angle of 30°, however, it generated a positive hazard warning in eight rounds out of ten, and two false hazard warnings were observed. Thus, it yielded a recall of 100% at 0° and 15° and 80% at 30°.

## 4. Discussion

### 4.1. Acceptability of Developed AI Smart Helmet-Based Personal PWS in the Field

Various sensing technologies have been used to develop PWSs for mining sites. Electromagnetic sensor technology is mainly used for ultra-close detection. Generating an electromagnetic field in a device to which a sensor is attached can detect objects within 10 m of the device or detect them on other equipment [32]. In the case of GPS, location and time information provided by the satellite navigation system are used. In accordance with the 2008 Standard Positioning Service (SPS) Performance Standard, the accuracy of the user range error (URE) is based on a measurement of approximately 7.8 m at a 95% confidence level [33]. Radar sensor technology generates a signal at a set frequency and measures the return echo and can be detected within 20–50 m of the surface mine environment. This technology can be used alone and can also be used with RFID tags. Microwave RFID is the most commonly used technique in proximity detection methods, which detects radio signals by sending them to RFID tags within the response range. It is possible to detect objects in the 20–100 m range in an open-air mine environment. Ultra-low frequency RFID also has a similar operating principle to microwave RFID. Wi-Fi technology converts identity information into a wireless signal using a wireless adapter and transmits a specific frequency to the router. Similar to microwave RFID, it is detected in the 20 to 100 m range in a surface mine environment [34]. Based on existing research cases, a minimum signal-receive distance of 20 m is expected to be appropriate on average. Therefore, it is appropriate to use the pixel value of the AI smart helmet system at 50 pixels in the field.

**Figure 14:**
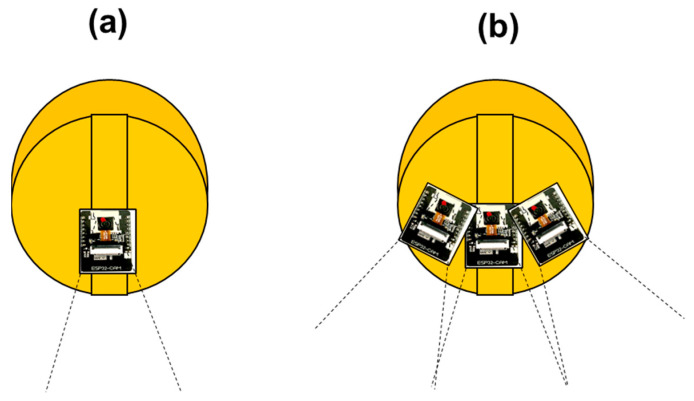
Comparison of recognition angles when there is one camera module (**a**) and three camera modules (**b**) on the AI Smart Helmet PWS system.

The developed AI smart helmet PWS is a system designed to ensure the safety of field workers. However, when the face angle between the camera module attached to the smart helmet and the recognition object is 30 degrees, there is a limitation that the recognition accuracy drops to 80%. Since this is a problem related to the safety of field workers, an accuracy of 80% is unacceptable in the field. Therefore, to improve this, we propose a complementary method in Figure 14. The proposed method is to compensate for the decrease in the recognition rate due to face angle by covering an angle that cannot be covered with one camera module with multiple camera modules.

### 4.2. Advantages of Developed AI Smart Helmet-Based Personal PWS

The AI smart helmet PWS has several noteworthy advantages. First, this system can address the limitations of conventional PWSs. In conventional systems, drivers need to repeatedly check their smartphones to receive proximity warnings, which reduces their concentration. Smart glasses cause discomfort due to sliding or when wearing regular glasses. Traditional smart helmets only generate proximity warnings for vehicles associated with Bluetooth beacons. In contrast, the AI smart helmet can provide both drivers and pedestrians visual proximity warnings without interrupting their work, thereby facilitating quick identification and evacuation of dangerous situations. It also provides accurate proximity warnings for all hazardous objects set in the app instead of only vehicles associated with the Bluetooth beacons. Second, the AI smart helmet is highly scalable; the system can be scaled by adding sensors as needed. Finally, it can be implemented and used in various environments, such as general urban centers, mining sites, and construction sites, at a relatively low cost. Given that the system uses Arduino and MIT App Inventor, configuring the components (microcontroller board and sensors) is relatively inexpensive. Therefore, regardless of the size of the applied domain, multiple sets of AI smart helmets can be deployed and used.

### 4.3. Limitations of Current Work and Future Research

In this study, the demonstrations and experiments of the smart helmet PWS were conducted on the roads on which real vehicles and trucks were moving. Due to the safety issue of the experimenter, there was a limit to controlling the vehicle and performing a large number of repeated experiments. Therefore, additional repeated experiments will be needed to secure a high reliability of the experimental results. The developed smart helmet PWS was tested in a surface environment with good lighting conditions. In order to confirm that the smart helmet PWS operates normally even in dark environments, repeated experiments in various light source environments will be required.

Using AI for decision-making carries a risk of misjudgment. There are five points that can give rise to AI risks (data difficulties, technology troubles, security snags, model misbehaviors, and human–machine interactions) [35]. Especially, misbehaving AI models used to recognize dangerous objects could be a critical problem for the smart helmet PWS. Therefore, the performance monitoring of AI models is necessary on a regular basis to reduce the risk of misjudgment. Also, more importantly, the developed smart helmet PWS does not completely eliminate the obligation of field workers to observe their surroundings.

Last, some sensors attached to the AI smart helmet such as cameras can potentially invade privacy. There is still insufficient research on the security and privacy of the data collected using the AI smart helmet. The proposed personal PWS collects and analyzes image data from the camera module to produce a hazard warning, but it cannot accurately generate hazard warnings for hazardous objects that approach from outside the camera’s field of view. Additionally, although various sensors can be attached to the AI smart helmet as needed, this increases the helmet’s weight and may make it inconvenient to wear. There is limited research on the associated discomfort and health effects of AI smart helmets. The following are requirements for future AI smart helmets:

1. Wearing comfort: Owing to the various sensors and microcontroller, it is relatively heavy compared to conventional hard hats. This can cause discomfort to motorcyclists and field workers. For the developed helmet to function as a wearable device, its weight must be reduced by using lightweight materials and components.
2. Human health and safety: Given that the developed AI smart helmet is worn on the head, the effects of electromagnetic fields emitted from sensors or microcontrollers on human health should be investigated.
3. Durability: Workers who wear AI smart helmets often work in very dusty and humid environments. Given that the microcontroller and camera module are exposed on the outside of the AI smart helmet, they are potentially vulnerable to the associated conditions. Research on the enhancement of the AI smart helmet’s durability is necessary to improve functionality even in poor work environments.
4. Accuracy: Depending on the camera module’s field of view, the developed AI smart helmet may not yield high accuracy. As such, to mitigate this issue caused by the camera module’s restricted field of view, research on the synthesis and analysis of image information from not one but multiple camera modules is needed.
5. Privacy and data security: The developed AI smart helmet collects a variety of information from the images of the attached camera module. However, this information can sometimes lead to privacy issues. For example, it is possible that unauthorized users may intercept information acquired by the AI smart helmet. Accordingly, continuous research on privacy and data security issues related to the use of this device is necessary.

## 5. Conclusions

In this study, a personal PWS was developed that generated visual and tactile proximity warnings to aid workers and operators. This was achieved by collecting image data from a camera module using an AI smart helmet, which was subsequently analyzed using a cloud server. To assess the personal PWS’s performance, the device’s recognition distance was evaluated according to the input minimum diagonal length and the hazard warning accuracy according to the face angle between the camera module and mining equipment on a straight road and in a mining site. According to the input minimum diagonal length, the average recognition distance was 63.25 m at 25 pixels, 24 m at 50 pixels, 14.25 m at 75 pixels, 11 m at 100 pixels, and 6.25 m at 125 pixels. This indicated that, as the minimum diagonal length increased, the AI smart helmet’s recognition distance decreased. The AI smart helmet exhibited a hazard warning accuracy of 100% when the face angle between the camera module and the mining equipment was 0° and 15°, and an accuracy of 80% when the angle was 30°.

In this study, we developed a smart helmet-based PWS that detects moving equipment such as trucks to prevent collision accidents. In future work, it would be interesting to consider other types of accidents or hazards using the smart helmet-based PWS. Another extension of the current work is to add various sensors, such as an alcohol sensor or a heart rate sensor, to check the condition of the workers, and a methane gas sensor or carbon monoxide sensor to monitor the environment.

## Figures and Tables

**Table 1:** Characteristics of sensor technology used for the development of PWSs (modified from [14]).

Sensing Technology	Frequency	Detection Range
Electromagnetic	70–140 kHz	10 m
Radar RFID	2.4 GHz	20–50 m
Ultra-High Frequency RFID	433 MHz, 860–960 MHz	20–100 m
Very Low Frequency RFID	<15 kHz	20–100 m
Wi-Fi	2.4 GHz	20–100 m
Bluetooth Low Energy	2.4 GHz	20–100 m

**Table 2:** Results of the recognition distance (m) of the AI smart helmet according to the input minimum diagonal length (pixels).

Signal Receive Distance (m)	Minimum Diagonal Length				
	25 Pixel	50 Pixel	75 Pixel	100 Pixel	125 Pixel
Mean	63.25	24	14.25	11	6.25
STD ^1^	1.21	2.11	2.37	2.11	2.12
Max ^2^	65	27.5	17.5	15	10
Min ^3^	62.5	20	10	7.5	2.5

^1^ Standard deviation; ^2^ Maximum value; ^3^ Minimum value.

**Table 3:** Results of the accuracy evaluation of the hazard warning of the AI smart helmet according to the face angle between the camera module and the mining equipment.

Type of Warning Alert	Angle between the Mining Equipment and the Pedestrian		
	0°	15°	30°
Trials	10	10	10
True positives	10	10	8
False negatives	0	0	2
Recall (%)	100	100	80

## Data Availability

Not applicable.

## References

[1] U.S. Bureau of Labor Statistics. https://www.bls.gov/iif/oshwc/cfoi/cftb0313.htm.

[2] Department of Mines, Industry Regulation and Safety. Government of Western Australia. http://www.dmp.wa.gov.au/Documents/Safety/MSH_R_VehicleCollisions.pdf.

[3] Ruff T.M. Recommendations for Evaluating and Implementing Proximity Warning Systems on Surface Mining Equipment. https://www.cdc.gov/niosh/mining/UserFiles/works/pdfs/2007-146.pdf.

[4] Ruff T.M. Overview of Proximity Warning Technology and Approaches. https://www.cdc.gov/niosh/mining/UserFiles/workshops/proximityworkshop2010/Ruff-NIOSH-PDWorkshop2010-508.pdf.

[5] Ruff T.M. Test Results of Collision Warning Systems for Surface Mining Dump Trucks. https://www.cdc.gov/niosh/mining/userfiles/works/pdfs/ri9652.pdf.

[6] Ruff T.M. Test Results of Collision Warning Systems for Surface Mining Dump Truck: Phase 2. https://www.cdc.gov/niosh/mining/UserFiles/works/pdfs/2001-100.pdf.

[7] Ruff T.M., Hession-Kunz D. (2001). Application of Radio-Frequency Identification Systems to Collision Avoidance in Metal/Nonmetal Mines. IEEE Trans. Ind. Appl..

[8] Schiffbauer W.H. (2002). An Active Proximity Warning System for Surface and Underground Mining Applications. Miner. Eng..

[9] Ruff T.M. Recommendations for Testing Radar-Based Collision Warning Systems on Heavy Equipment. https://www.cdc.gov/niosh/mining/UserFiles/works/pdfs/ri9657.pdf.

[10] Ruff T.M., Holden T.P. (2003). Preventing Collisions Involving Surface Mining Equipment: A GPS-Based Approach. J. Saf. Res..

[11] Ruff T.M. (2004). Advances in Proximity Detection Technologies for Surface.

[12] Ruff T.M. (2006). Evaluation of a radar-based proximity warning system for off-highway dump trucks. Accid. Anal. Prev..

[13] Baek J., Choi Y. (2018). Bluetooth-Beacon-Based Underground Proximity Warning System for Preventing Collisions inside Tunnels. Appl. Sci..

[14] Lee C., Suh J., Baek J., Choi Y. (2017). Review of Collision Avoidance Systems for Mine Safety Management: Development Status and Applications. Tunn. Undergr. Space.

[15] Kim Y., Baek J., Choi Y. (2021). Smart Helmet-Based Personnel Proximity Warning System for Improving Underground Mine Safety. Appl. Sci..

[16] ELOshield by ELOKON. https://www.elokon.com/en-EN/intralogistics/eloshield-proximity-detection.html.

[17] Joy Smartzone Proximity System. https://mining.komatsu/technology/proximity-detection/smartzoneproximity-detection.

[18] Smartzone Proximity System by JoyGlobal. https://mining.komatsu/docs/default-source/non-productdocuments/technology/proximity-detection/smartzone-pamphlet.pdf?sfvrsn=56060a6b_46.

[19] Proximity Detection for Underground Coal Mines. https://www.strataworldwide.com/sites/default/files/platform/brochure/StrataProximity-Coal-Mining-US_2018.pdf.

[20] Jobes C., Carr J., DuCarme J. (2012). Evaluation of an advanced proximity detection system for continuous mining machines. Int. J. Appl. Eng. Res..

[21] Proximity Detection System by NAUTILUS International. http://www.nautilus-intl.com/proximity-detection/nautilus-coal-buddy-operators-proximity-detection-system-for-underground-coal-mines-operating-in-an-explosive-methanegas-environment-class-i-div-ii/.

[22] Proximity Warning System by Sensorzone. https://globalsurvey.co.nz/wp-content/uploads/2016/06/A4-Brochure-final.pdf.

[23] SiteZone PWS. https://proximitywarning.com/product-services/sitezone-proximity-warning-system/.

[24] Hines K.P. (2016). Exploration of Alerting Methods on Vest-Worn Systems. Master’s Thesis.

[25] Sakhakarmi S., Park J., Singh A. (2021). Tactile-based wearable system for improved hazard perception of worker and equipment collision. Autom. Constr..

[26] Baek J., Choi Y. (2020). Smart Glasses-Based Personnel Proximity Warning System for Improving Pedestrian Safety in Construction and Mining Sites. Int. J. Environ. Res. Public Health.

[27] ESP 32-CAM Specification. https://randomnerdtutorials.com/esp32-cam-troubleshooting-guide/.

[28] ETRI Public AI open API platform. https://aiopen.etri.re.kr.

[29] Bae S.H., Lee Y., Jo Y., Bae Y., Hwang J.W. (2017). Rank of experts: Detection network ensemble. arXiv.

[30] Arduino UNO Board Specification. https://store.arduino.cc/usa/arduino-uno-rev3.

[31] HC-06 Bluetooth Module Specification. https://www.etechnophiles.com/hc06-pinout-specifications-datasheet/.

[32] Jobes C., Carr J., DuCarme J., Patts J. Determining Proximity Warning and Action Zones for a Magnetic Proximity Detection System. Proceedings of the 2011 IEEE Industry Applications Society Annual Meeting.

[33] Brent A.R., Audric T., Nicholas B. An Analysis of Global Positioning System (GPS) Standard Positioning System (SPS) Performance for 2017. https://www.gps.gov/systems/gps/performance/2017-GPS-SPS-performance-analysis.pdf.

[34] Dunn M., Hargrave C., Vowles M. Proximity Detection Device Interoperability. https://publications.csiro.au/rpr/download?pid=csiro:EP178511&dsid=DS1.

[35] Cheatham B., Javanmardian K., Samandari H. Confronting the Risks of Artificial Intelligence. https://www.mckinsey.com/capabilities/quantumblack/our-insights/confronting-the-risks-of-artificial-intelligence.

